# Tensile properties of the rectal and sigmoid colon: a comparative analysis of human and porcine tissue

**DOI:** 10.1186/s40064-015-0922-x

**Published:** 2015-03-26

**Authors:** Michael B Christensen, Kevin Oberg, Jeffrey C Wolchok

**Affiliations:** Keck Center for Tissue Engineering, Department of Bioengineering, University of Utah, 36 S Wasatch Dr. Rm 3100, Salt Lake City, UT 84112 USA; Department of Biomedical Engineering, University of Arkansas, 120 Engineering Hall, Fayetteville, AR 72701 USA

**Keywords:** Colorectal tissue, Mechanical properties, Tensile properties, Porcine Tissue, Human tissue

## Abstract

For many patients, rectal catheters are an effective means to manage bowel incontinence. Unfortunately, the incidence of catheter leakage in these patients remains troublingly high. Matching the mechanical properties of the catheter and the surrounding tissue may improve the catheter seal and reduce leakage. However, little data is available on the mechanical properties of colorectal tissue. Therefore, our group examined the mechanical properties of colorectal tissue obtained from both a common animal model and humans. Uniaxial tension tests were performed to determine the effects of location, orientation, and species (porcine and human) on bowel tissue tensile mechanical properties. Bowel tissue ultimate strength, elongation at failure, and elastic modulus were derived from these tests and statistically analyzed. Ultimate tensile strength (0.58 MPa, 0.87 MPa), elongation at failure (113.19%, 62.81%), and elastic modulus (1.83 MPa, 5.18 MPa) for porcine and human samples respectively exhibited significant differences based on species. Generally, human tissues were stronger and less compliant than their porcine counterparts. Furthermore, harvest site location and testing orientation significantly affected several mechanical properties in porcine derived tissues, but very few in human tissues. The data suggests that porcine colorectal tissue does not accurately model human colorectal tissue mechanical properties. Ultimately, the tensile properties reported herein may be used to help guide the design of next generation rectal catheters with tissue mimetic properties, as well as aid in the development of physical and computer based bowel models.

## Introduction

Health care providers responsible for the treatment of critical care patients or long term nursing home residents are frequently faced with the challenge of managing fecal incontinence (Bliss et al. 2007; Borrie and Davidson 1992; Peet et al. 1995). If not properly managed, persistent fecal contamination can lead to incontinence associated dermatitis (Long et al. 2011; Zimmaro Bliss et al. 2006) which can lead to breakdown of the skin within the perineal of perigential areas. Breakdown of the protective barrier provided by the skin makes patients more susceptible to complications that include pressure ulceration (Allman et al. 1995; Halfens et al. 2000; Keller et al. 2002; Long et al. 2011; Maklebust and Magnan 1994; Theaker et al. 2000) and fecal carriage of antibiotic-resistant bacteria, leading to infection (Bliss et al. 2000; Bonomo et al. 2003; Mirelis et al. 2003; Valverde et al. 2004).

Historically, the management of fecal incontinence has focused on the containment of stool using absorbent pads and pouches. More recently, the use of rectal catheters has been adopted as the preferred bowel management system. Similar in concept to the urinary catheter, rectal catheters can reduce the incidence of fecal contamination by directly channeling stool from the colon to a collection bag. When compared to pads, the use of rectal catheters to mange fecal incontinence has been shown to reduce both the incidence of incontinence associated dermatitis and the overall cost of treating these patients (Kowal-Vem et al. 2009; Pittman et al. 2012).

Despite the benefits of rectal catheter use, incidents of rectal leakage are not uncommon. One recent study determined that the number of unscheduled bedding or dressing changes due to fecal leakage around rectal catheters was higher than one per day (Kowal-Vem et al. 2009). To improve clinical performance, next generation rectal catheters with an improved seal between the catheter and the rectal tissue need to be designed. Matching the mechanical properties of the rectal catheter to that of the surrounding tissue may be advantageous in improving the seal. However, publications detailing the mechanical properties of colorectal tissue in human are limited.

Traditionally, porcine tissue has been used for the modeling of various human tissues in medicine due to both size and functional similarities, as well as the ease of procurement. Specifically, it has been suggested that human and pig colons have similar morphologies (Kararli 1995). Additionally, a number of studies describing the biomechanical properties of porcine colorectal tissue already exist (Carniel et al. 2014a; Carniel et al. 2014b; Qiao et al. 2005). Potential similarities and the existence of biomechanical data might make porcine tissue an ideal model that could be used to guide the design of next generation rectal catheters. However, although anatomically similar, it is unclear whether similarities extend to mechanical properties. Towards this end, the goal of this study was the measurement of both porcine and human colorectal tissue tensile properties. This information could be helpful in the development of next generation rectal catheters with tissue mimetic properties, as well as computer modeling of colorectal tissues. This data will also help determine the validity of using porcine rectal tissue as a test platform for human rectal tissue.

## Methods

### Sample collection and preparation

Approximately 30 cm of porcine bowel tissue (Yorkshire hogs aged 3–6 months), extending from the anus to the sigmoid colon, was procured (N = 17) from a commercial slaughterhouse (Tooele Valley Meats, Grantsville, UT) immediately after slaughter. Similarly sized male and female human bowel tissue samples (Table 1) were procured (N = 11) from a commercial tissue donation center (Science Care, Phoenix, AZ, www.sciencecare.com). Human tissue was frozen prior to procurement. All tissue samples were stored at −20°C after procurement and prior to mechanical testing.

**Table 1:** **Human tissue donor demographics (average ± standard deviation)**

	**Male (n = 5)**	**Female (n = 6)**	**All (n = 11)**
Age (years)	70.8 ± 13.6	64.2 ± 12.2	67.2 ± 13.7
Height (in)	70.8 ± 2.77	63.5 ± 3.3	66.8 ± 4.8
Weight (lbs)	185 ± 53.4	233.8 ± 66.8	211.6 ± 63.4
BMI	25.8 ± 7.1	40.1 ± 12.1	34 ± 12.5

**Figure 1:**
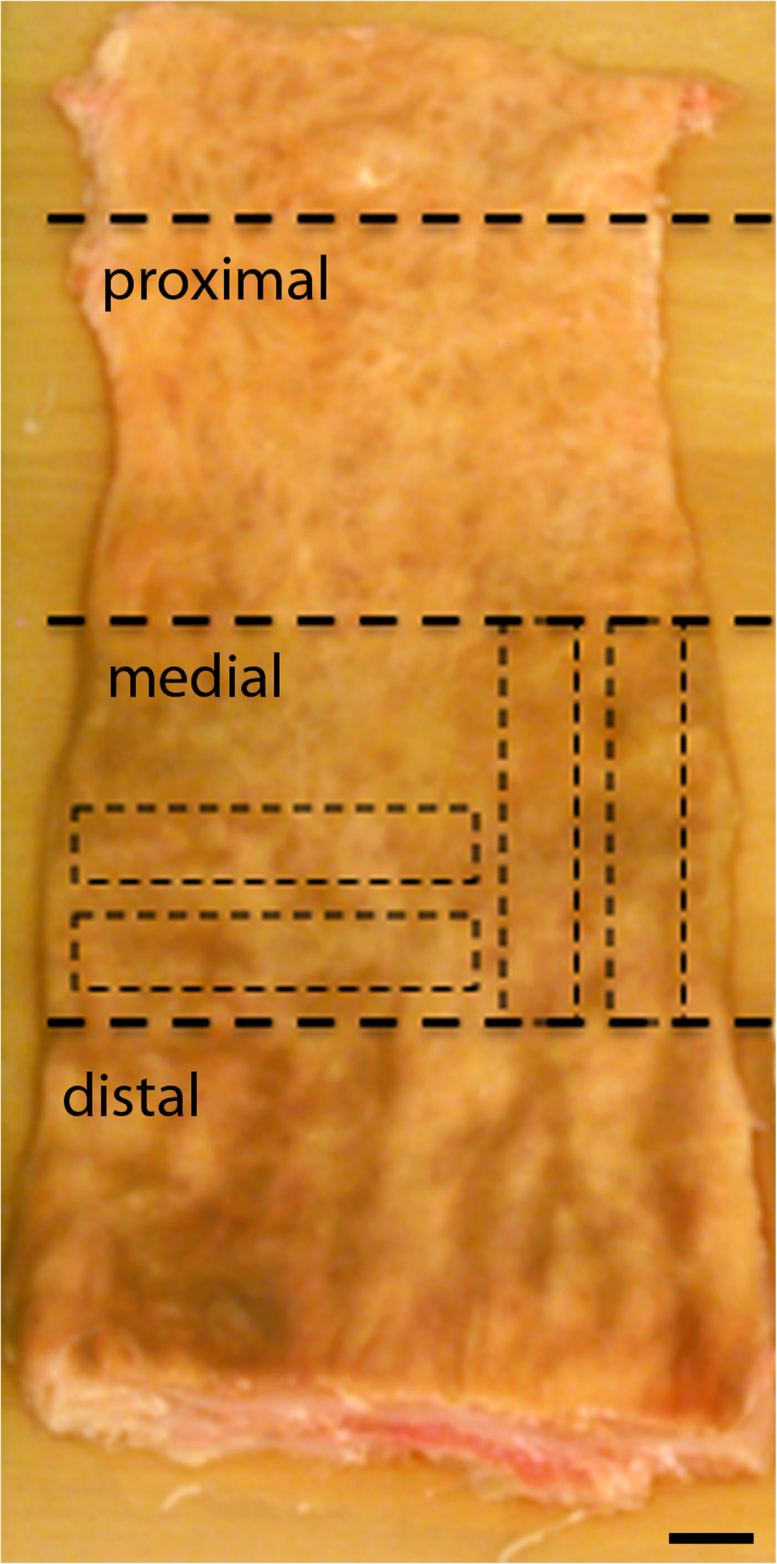
**Dissected porcine colon illustrating the approximate location of distal, medial, and proximal sections (thick dashed lines).** Within each section, two longitudinal and two circumferential test sections (dashed line boxes, shown for medial section only) were harvested. Scale bar = 1 cm.

**Figure 2:**
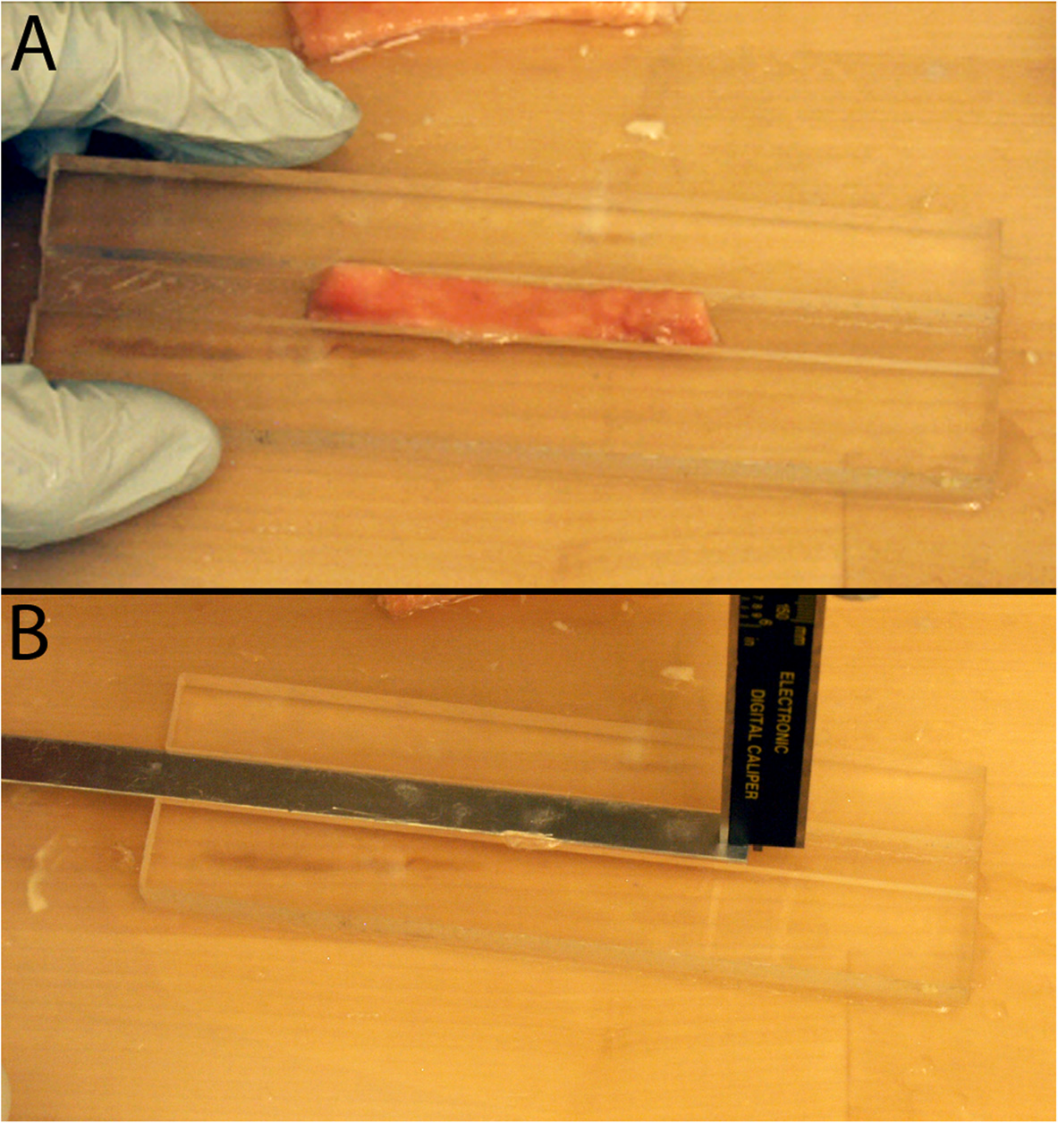
**For measurement of average thickness for each section, tissue was placed within a width constraining trough (A) and a thin piece of metal placed over the tissue so that it was laying flat (B).** Digital calipers were then used to measure the distance from the bottom of the trough to the bottom of the metal for each sample.

Tissue specimens were thawed at room temperature in preparation for mechanical testing. Once thawed, the tissue was thoroughly rinsed, the anus was dissected from the end of the intestines, and a longitudinal incision was made beginning at the distal end of the colon (the dentate line) and continued proximally. Excess fat and connective tissue were removed, whenever possible, from the extraluminal wall of the rectal tissue. The bowel was laid open and beginning just proximal to the dentate line, the tissue was divided into three 5-7 cm sections encompassing the distal, medial, and proximal regions of the colon (Figure 1). Four samples (two circumferential and two longitudinal) of 1 cm x 5 cm in size were prepared from each region using a custom made punch. A total of 12 samples were tested for each animal or human donor. For measurement of average thickness for each section, tissue was placed within a width constraining trough 1 cm wide and a thin piece of metal was placed over the tissue so that it was laying flat. Digital calipers were then used to measure the distance from the bottom of the trough to the bottom of the metal for each sample (Figure 2). Prepared samples were maintained in saline saturated gauze prior to testing.

### Tensile testing

Uniaxial tensile testing was accomplished with the aid of a computer controlled material testing system (Model 3342, Instron, Norwood, MA) incorporating a 50 N load cell. Manufacturer software (Bluehill® Lite, Instron, Norwood, MA) was used to control strain rate and record force/elongation data. Samples were aligned in the direction of loading and secured at each end using hand tightened serrated grips. No slipping of the tissue during any test was observed either visually or within the data. Rectangular samples were used based on preliminary data from a separate set of porcine colorectal tissue using identical tensile settings which showed consistent breakage near the midpoint of the tissue (data not shown). Therefore, to ensure consistency and ease in tissue preparation, rectangular, rather than traditional dogbone-shaped, specimens were employed. A gauge length of 2 cm was used for all samples. Prior to tensile testing, samples were pre-conditioned using ten cycles of 20% strain to establish a uniform loading history for all samples. Immediately following pre-conditioning, each sample was loaded to failure at a quasi static strain rate of 1%/sec (Hardcastle and Mann 1968). Raw data were collected at a sampling rate of 10Hz. For each tissue sample, engineering stress versus strain curves were generated from load and elongation data. From these curves, ultimate tensile strength, tan modulus, and elongation at failure were calculated. Ultimate strength was calculated as the maximum stress attained by the sample prior to failure. Percent elongation at failure was calculated as the percent elongation attained at the point of maximal stress. The elastic modulus was calculated as the slope of a linear curve fit to the stress–strain region extending from the end of the toe-in region to the point of maximum stress.

### Data analysis

For statistical comparisons, mean and standard deviation values for all mechanical testing measures (ultimate strength, percent elongation at failure, and elastic modulus) were calculated for each location, orientation, and species. Outlier data were identified using Chauvenet’s criteria and removed from data sets. Location differences within each species were tested using paired t-tests, with a Bonferroni-adjusted significance level less than 0.0167 being required to reach significance at the p = 0.05 level. Orientation differences within each species were also compared using a paired *t*-test. Cross-species differences were tested using an unpaired *t*-test assuming unequal variance. Sex differences in the human data were tested using an unpaired *t*-test assuming equal variance. Correlations between human donor age or BMI and mechanical measures were testing using regression analysis. P-values less than 0.05 were considered significant. All data is represented as mean ± SEM.

## Results

### Porcine versus human

**Figure 3:**
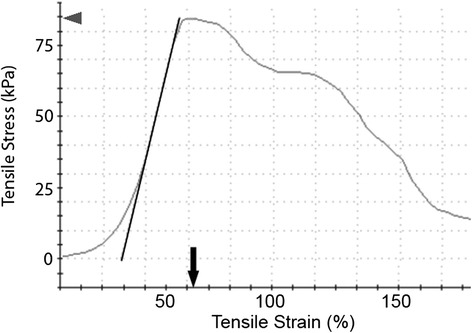
**Representative stress–strain curve collected from human rectal tissue.** Stress represents the load per cross-sectional area being applied to the sample. Strain represents the elongation of the specimen relative to its initial length (2 cm). Ultimate strength was calculated as the maximum stress (arrowhead) applied to a specimen, and in all cases for this study was the point at which the tissue substantially tore. Elongation at break (arrow) is the strain achieved by the sample at the point of ultimate stress. Modulus was calculated as the slope of the linear region within the stress/strain curve.

**Figure 4:**
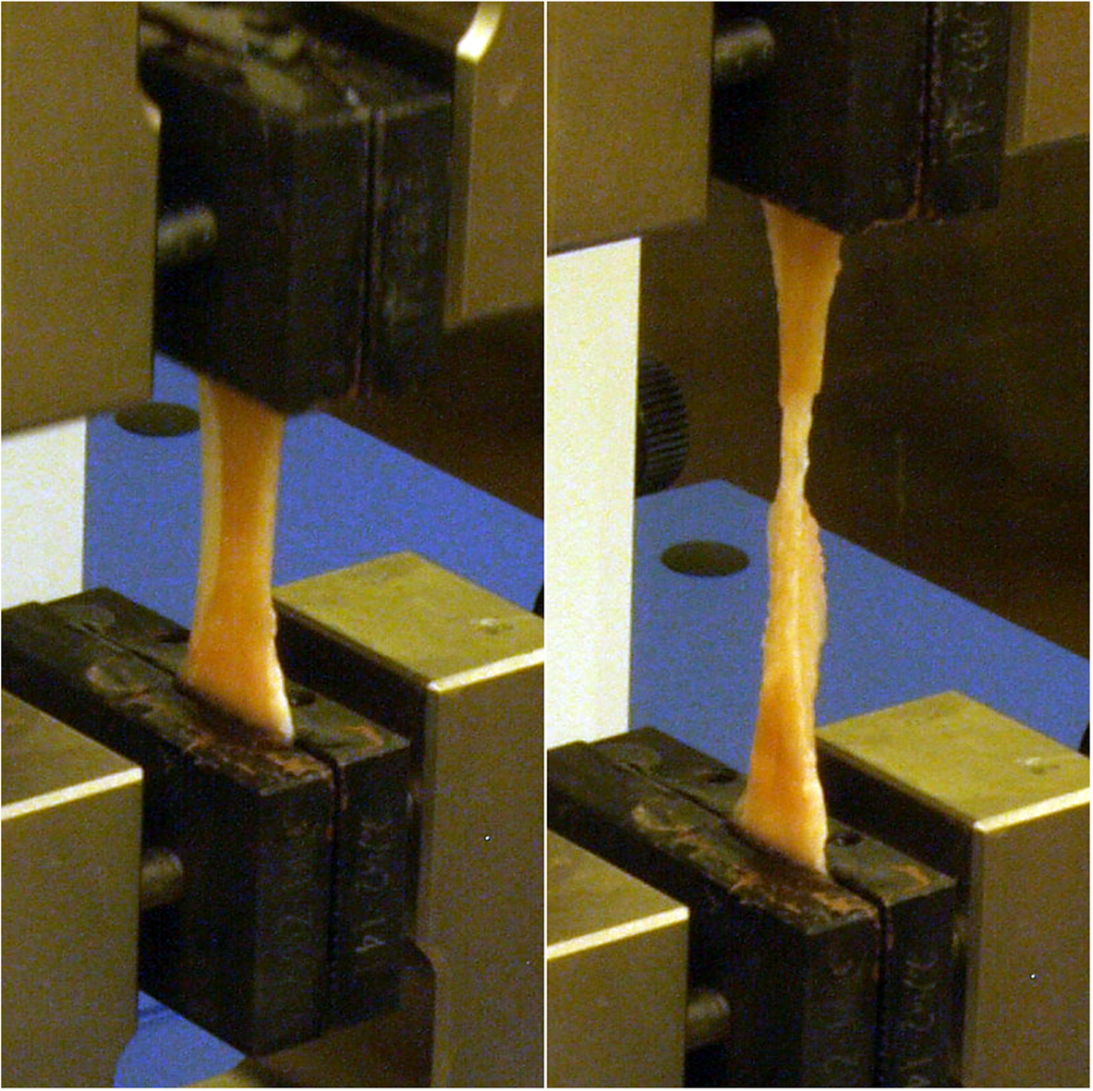
**Representative images of tissue deformation during tensile testing.** Tissue was placed between two serrated grips, with an initial gauge length of 2 cm, and pulled at a constant rate of 1%/sec (Left). All tissue samples tested thinned mid-substance before ultimately failing through progressive tearing at the same location (Right).

Both human and porcine stress versus strain curves were characterized by a short toe in region extending out to about 5% strain, followed by a linear rise in stress with strain that continued out the point of ultimate strength, all followed by a extended “plastic-like” deformation region characterized by irreversible tissue failure with progressive tearing and a reduction in stress (Figure 3). Tearing during testing originated near the center of the sample, not at the gripped ends (Figure 4). Average tissue cross-sectional area for porcine specimens was 20.90 mm^2^ while that for human specimens was 16.21 mm^2^. Average tensile testing data measured from porcine and human tissue samples are summarized in Tables 2 and 3 respectively. All data is represented as average with (standard error). All statistical comparisons, representing comparisons between species, orientation, and harvest location, are summarized in Table 4.

**Table 2:** **Porcine rectal tissue mechanical properties**

**Ultimate strength: MPa**	**Circumferential**		**Longitudinal**		**All orientations**	
Distal	0.54	(0.07)	0.62	(0.07)	0.58	(0.05)
Medial	0.47	(0.04)	0.47	(0.04)	0.47	(0.03)
Proximal	0.58	(0.08)	0.59	(0.09)	0.58	(0.06)
All Locations	0.51	(0.03)	0.63	(0.05)	0.58	(0.03)
**Elongation at failure: %**	Circumferential		Longitudinal		All orientations	
Distal	141.84	(13.62)	149.28	(13.67)	145.56	(9.38)
Medial	95.47	(7.76)	122.22	(8.91)	108.85	(6.20)
Proximal	90.86	(6.38)	103.89	(8.20)	97.37	(5.17)
All Locations	106.27	(5.61)	120.25	(5.63)	113.19	(4.00)
**Elastic modulus: MPa**	Circumferential		Longitudinal		All orientations	
Distal	0.92	(0.22)	0.86	(0.18)	0.89	(0.14)
Medial	2.00	(0.40)	1.45	(0.32)	1.74	(0.26)
Proximal	2.10	(0.41)	2.12	(0.47)	2.11	(0.30)
All locations	1.87	(0.23)	1.79	(0.24)	1.83	(0.16)

Values shown are mean (SEM).

**Table 3:** **Human rectal tissue mechanical properties**

**Ultimate strength: MPa**	**Circumferential**		**Longitudinal**		**All orientations**	
Distal	0.86	(0.10)	0.86	(0.09)	0.86	(0.07)
Medial	0.86	(0.11)	0.97	(0.08)	0.92	(0.06)
Proximal	0.77	(0.11)	0.88	(0.12)	0.82	(0.08)
All Locations	0.84	(0.06)	0.93	(0.05)	0.87	(0.04)
**Elongation at failure: %**	Circumferential		Longitudinal		All orientations	
Distal	61.43	(0.63)	61.70	(0.72)	61.54	(0.44)
Medial	60.72	(0.58)	62.37	(0.79)	61.31	(0.49)
Proximal	63.43	(1.02)	64.72	(1.50)	64.08	(0.86)
All Locations	61.74	(0.47)	64.31	(0.76)	62.81	(0.44)
**Elastic modulus: MPa**	Circumferential		Longitudinal		All orientations	
Distal	5.20	(0.55)	4.87	(0.65)	5.04	(0.40)
Medial	6.48	(0.82)	5.34	(0.50)	5.91	(0.48)
Proximal	3.88	(0.34)	4.87	(0.40)	4.35	(0.28)
All locations	5.41	(0.39)	5.05	(0.29)	5.18	(0.24)

Values shown are mean (SEM).

**Table 4:** **Statistical comparison p-values**

	**Ultimate strength**	**Elongation at failure**	**Elastic modulus**
**Porcine data**			
Distal vs. Medial	0.167	*9.46E-05*	*0.010*
Distal vs. Proximal	0.731	*2.30E-07*	*0.002*
Medial vs. Proximal	0.200	0.024	*0.011*
Circumferential vs. Longitudinal	*0.001*	*0.003*	0.669
**Human data**			
Distal vs. Medial	0.415	0.444	0.130
Distal vs. Proximal	0.759	0.023	0.164
Medial vs. Proximal	0.054	*0.011*	0.079
Circumferential vs. Longitudinal	0.179	0.087	0.538
**Collective data**			
Human vs. Porcine	*2.04E-08*	*2.28E-22*	*2.75E-21*

The average ultimate strength, elongation at failure, and elastic modulus, calculated as the average across all locations and orientations, were significantly different between human and porcine tissue samples. Human tissue was more than twice as stiff as porcine tissue. Specifically, the average porcine and human rectal tissue elastic modulus values were 1.83 ± 0.16 MPa and 5.18 ± 0.24 MPa respectively. Both porcine and human bowel tissue samples elongated substantially before failing, with porcine tissue exhibiting approximately twice as much elongation as human tissue. Average strain at failure for porcine tissue was measured at 113.2 ± 4.0% while human tissue strain at failure was measured at 62.8 ± 0.4%. Ultimate strength values were also significantly different between species, with human tissue exhibiting higher average ultimate strengths. Porcine tissue ultimate strength was 0.58 ± 0.03 MPa while human tissue was 0.87 ± 0.04 MPa. Generally human tissue was stronger, stiffer, and less compliant than porcine tissue, with ultimate strength, elastic modulus, and elongation at failure values being 150%, 283%, and 56% of those measured from porcine tissue respectively. No differences in any mechanical measures in the human data were detected based on sex. Additionally, no correlation between mechanical measures and age or BMI in the human data was found (data not shown).

### Location and orientation

For porcine tissue samples, statistically significant differences were detected based on both harvest location and orientation. When harvest location was examined, we observed that tissue samples collected from the distal region closest to the anus were generally less stiff and more compliant than tissues collected from the medial and proximal regions. The elongation at failure for distal tissue samples was 52% greater than proximally collected samples and 34% greater than medial samples. Similarly, the modulus of elasticity measured for distal samples was approximately half that of proximal (42%) and medial sections (51%). Yet, while modulus and strain at failure were affected by harvest location, the ultimate strength was not. The average ultimate strength of samples collected from distal sections were within 10% and 1% of medial and proximal samples respectively.

When sample orientation was examined, we observed that longitudinally oriented porcine tissue samples were generally stronger and more compliant than circumferential samples. Longitudinal tissue ultimate strength and strain at failure was 25% and 15% greater than circumferential samples respectively. Both differences were statistically significant. Alternatively, the elastic modulus was not significantly affected by sample orientation. Longitudinally oriented tissue elastic modulus was 4% less than circumferential values, a non-significant difference.

While porcine colorectal tissue statistical analysis revealed multiple differences in tensile mechanical properties based on harvest site location and orientation, the testing of human tissue samples did not reveal similar differences. No differences in strength, elasticity, or strain at failure were measured between longitudinal and circumferential samples. Similarly, strength, elasticity, and strain at failure were generally uniform regardless of the harvest site location (distal, medial, proximal). The only statically significant difference detected was strain at failure, which was greater in samples collected from medial sections when compared to proximal samples, although the difference was only 5%. Overall, human colorectal tissue tensile properties were not influenced by harvest site location or orientation

## Discussion

The mechanical properties of porcine and human bowel tissue were obtained via uniaxial tensile testing. Data were collected from samples oriented in both the longitudinal and circumferential directions as well as from tissues harvested from distal, medial, and proximal regions. While the general assumption at the beginning of this study was that circumferential sections would have a lower modulus than longitudinal sections, owing to the circumferential dilation of bowel tissue during digestion and stool storage, statistical comparisons in both porcine and human tissue suggests that no such differences between orientations exists. Furthermore, porcine data suggests that certain parameters, primarily elongation at failure, do have some dependency on location along the bowel. However, few of these dependencies were replicated in the human data, suggesting that our findings might be species dependant rather than conserved across species in the same tissue type.

Porcine and human tissue samples exhibited significant differences from each other for all tested parameters, with elongation at failure being lower in human samples, and ultimate strength and elastic modulus being higher in human samples. The exact cause for these differences is unclear. However, differences in mechanical properties may be due to minor differences in digestive mechanisms or system composition. For instance, it has been shown that the microbiota in fecal matter from pigs and humans are significantly different (Furet et al. 2009; Lee et al. 2011). Studies have also suggested more efficient breakdown of certain whey proteins by porcine gastrointestinal enzymes compared with those found in humans (Eriksen et al. 2010). Slight anatomic differences in the stomach between species also exist (Kararli 1995). These differences may contribute to known differences in defecation frequency between species (Heaton et al. 1992; Z’Graggen et al. 1999), which may in turn lead to the mechanical differences observed here.

It is also interesting to note the variability in data sets. Porcine data tended to be more variable than human data. For ultimate strength, both porcine and human data had standard errors that averaged about 10% of the mean. For elongation at failure, porcine samples had standard errors that averaged 7% of the mean, while human data had little variability, with standard errors averaging only 1% of the mean. For elastic modulus, porcine samples had about twice the variability of human samples, with standard errors averaging 17% and 9% of the means respectively. Therefore, even if porcine tissue were an acceptable alternative to human tissue for mechanical property measurements, larger variability in porcine data sets would likely exist.

Few biomaterial studies evaluating the mechanical properties of bowel tissue in either hogs or humans have been conducted (Carniel et al. 2014a; Carniel et al. 2014b; Egorov et al. 2002; Qiao et al. 2005; Watters et al. 1985). Qiao et al. published modulus values for porcine bowel tissue as 59.9kPa for longitudinal sections and 147kPa for circumferential sections (Qiao et al. 2005). Both of these values are 1–2 orders of magnitude lower than the values we collected. While pre-conditioning of our samples was similar to the method used by Qiao et al., strain rates used in that study were not published and differences between the studies may account for observed differences. While our measured porcine properties differ from the previously published values, the human tissue data is in good agreement with published tensile strength (0.65–0.95 MPa) and elongation at break (136–173%, reported as L/L_0_) values (Egorov et al. 2002; Watters et al. 1985). Furthermore, our data also suggests that significant differences do not exist between human circumferential and longitudinal mechanical properties, which was unexpected but is in agreement with published data (Egorov et al. 2002). When taken as a whole, the valuable contribution of our study to the existing published colon data is the combined compilation of orientation, location, and species data for colon tissue using uniform testing conditions. While past studies have looked at location (Watters et al. 1985) or orientation (Egorov et al. 2002), we are the first to measure and report on all three parameters. Additionally, our data is the first to indicate that porcine rectal tissue may not provide an ideal model for human tissue, a finding that could influence future pre-clinical studies.

Comparison of bowel tissue mechanical properties to other tissues provides some insights into the bowels function. When compared to high load bearing musculoskeletal tissues, like tendon and ligament, the mechanical properties of bowel tissues are substantially lower. For instance, the elastic modulus of the human patellar tendon is on the order of 500 MPa, or approximately two orders of magnitude greater than our measured values for human bowel tissue (5.35 MPa) (Hashemi et al. 2005). Yet when compared to tissues that undergo significant dilation and expansion, such as lung and stomach tissue, our measured elastic modulus values for bowel tissues are similar in magnitude (Lim et al. 2009; Liu and Tschumperlin 2011). These comparisons suggest that a suitable range of mechanical property values, particularly elastic modulus values between 1-10 MPa, exists for mammalian tissues that undergo significant dilation.

In this study all tissues were collected fresh and then frozen to −20°C for storage. The use of cold storage could potentially cause structural and biochemical changes that would influence bowel tensile properties. For example, it has been reported that cold storage decreases the soluble and insoluble collagen content within cold stored aortic tissues (Venkatasubramanian et al. 2006). Whether similar biochemical changes occur within bowel tissues following cold storage was not examined in this study, but could be assumed. A reduction in collagen content would likely decrease bowel tensile properties including the elastic modulus and ultimate strength. However, published research suggests that the effect is most pronounced at the low-strain toe in region of the load deformation curve and less significant at the higher elongation values reported in this study.

In order to improve clinically used rectal catheters and decrease leakage rates, we believe the compliance of the catheter should match that of the tissue. Here, we measured the elastic modulus of bowel tissue from hogs and humans to be in the range of 1-6 MPa. Rectal catheters are typically composed of silicone, which has an elastic modulus around 10-12 MPa (Cervera et al. 1989; Crnich et al. 2005). Increased compliance of the catheter would likely lead to a better seal and mitigate the frequency of leaks. The data could also be used to help guide the development of physical models. Mechanically realistic bowel models could be used as hands on training aids for health care providers learning effective placement and maintenance of rectal catheters. As the baby boomer population continues to age, critical care needs for this population will also continue to increase. As a result, the need to train critical care health care providers in the proper use of rectal catheters will increase as well. It is generally difficult to provide live human training experiences for student nurses; as a result it is common during the early training period to utilize realistic anatomical models (Lateef 2008). We believe a mechanically realistic human bowel model could have significant clinical utility as a training tool.

## Conclusion

This focused biomechanics study provides a comprehensive set of both porcine and human bowel tissue tensile mechanical properties, encompassing testing of tissues segregated by both harvest location and testing orientation. This study provides valuable knowledge about the mechanical properties of bowel tissue from hogs and humans which could aid the broader scientific community in the design of next generation rectal catheters as well as improvements to colorectal tissue mathematical or physical models. Importantly, this study shows through comparison of porcine to human data, that porcine tissue does not provide an accurate approximation to human tissue when considering ultimate stress, elongation at break, or modulus. As a goal for future work, it may be important to know how the mechanical properties vary within the human population as a result of patient age, weight, and sex. Future studies, investigating the affect of these parameters on bowel tissue mechanical properties could reveal subtle differences between patient groups that would help further guide the development of colorectal models or medical devices.
